# Agenesis of the Gallbladder in Monozygotic Twin Sisters

**DOI:** 10.1155/2016/1053138

**Published:** 2016-01-26

**Authors:** Koki Hoshi, Atsushi Irisawa, Goro Shibukawa, Akane Yamabe, Mariko Fujisawa, Ryo Igarashi, Ai Sato, Takumi Maki

**Affiliations:** Department of Gastroenterology, Aizu Medical Center, Fukushima Medical University, 21-2 Maeda, Tanisawa, Kawahigashi, Aizuwakamatsu 969-3492, Japan

## Abstract

Agenesis of the gallbladder, a rare anomaly, is generally regarded as an organogenic failure. Several reports suggest that this congenital defect is inherited but that supposition remains controversial. We described agenesis of the gallbladder in identical twins. A 21-year-old female presented with a history of acute pain in the epigastrium and right hypochondrium. Various imaging modalities showed “gallbladder agenesis.” Moreover, her older identical twin sister had also no visualized gallbladder in imaging modalities. This case report strongly suggested that agenesis of the gallbladder would be caused by a genetic abnormality.

## 1. Introduction

Agenesis of the gallbladder, a rare anomaly, is generally regarded as an organogenic failure. Several reports suggest that this congenital defect is inherited but that supposition remains controversial. Herein, we describe the agenesis of the gallbladder in identical twins.

## 2. Case Presentation

**Figure 1:**
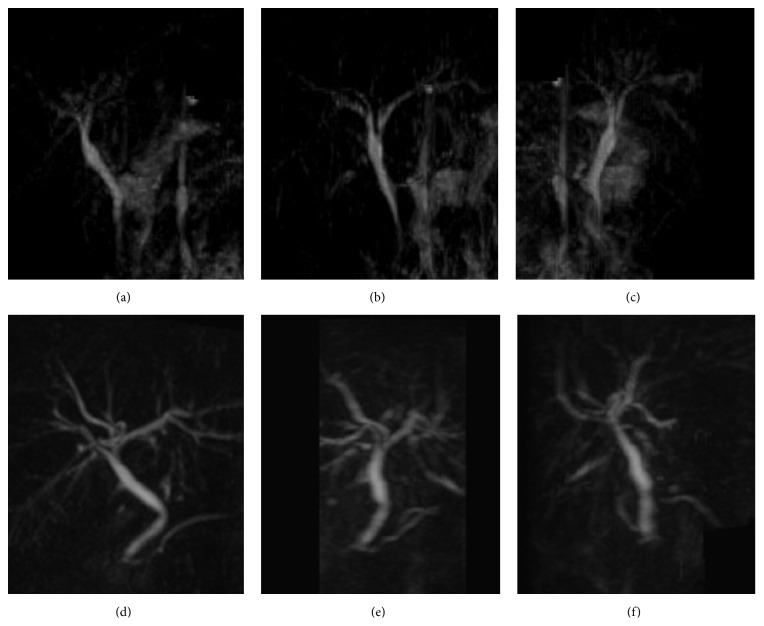
MRCP images of twin older sisters. Judging from various angles, the gallbladder was not detected on MRCP. ((a)–(c)) MRCP images of twin older sister. ((d)–(f)) MRCP images of twin younger sister.

A 21-year-old female was referred to our hospital. She presented with a history of acute pain in the epigastrium and right hypochondrium for the prior 5 days. She had never experienced a similar episode. Relevant hematological investigations yielded the following data: total bilirubin, 0.4 mg/dL; direct bilirubin, 0.1 mg/dL; alkaline phosphatase, 223 IU/L; GGTP, 13 IU/L; AST, 17 IU/L; and ALT, 12 IU/L. Ultrasonography (US) showed that the liver was normal; the common bile duct (CBD) was 6 mm and not dilated. Despite our best efforts, the gallbladder could not be localized. Endoscopic ultrasonography (EUS) revealed that the CBD was not dilated, but the gallbladder could not be localized. Contrast enhanced computed tomography (CT) and magnetic resonance cholangiopancreatography (MRCP) showed that the gallbladder was not visualized without abnormalities of intrahepatic/extrahepatic bile duct (Figures 1(a)–1(c)). Although we did not perform any additional examination such as endoscopic retrograde cholangiopancreatography (ERCP) or angiography because of her age and disappearance of symptoms, we diagnosed “gallbladder agenesis” based on the findings of various imaging modalities.

This patient's slightly older identical twin sister was encouraged to visit our hospital. She was asymptomatic. US and MRCP showed no visualized gallbladder (Figures 1(d)–1(f)). These modalities revealed a nondilated bile duct and lack of a cystic duct. Finally, we diagnosed the same gallbladder agenesis as that present in her younger identical twin sister. Neither parent had any biliary system abnormality.

## 3. Discussion

Agenesis of the gallbladder, an extremely rare condition with incidence of 0.01%–0.02%, is characterized by the absence of the gallbladder without atresia of the extrahepatic biliary system [1]. The pathogenesis of agenesis of the gallbladder is known to be related to embryonic development. During the fifth week of intrauterine life, the gallbladder and cystic duct start to develop as a bud from the CBD. Agenesis of the gallbladder occurs because of failure of the bud to proliferate or canalize. In earlier studies of the literature, although the male/female ratio has been equal in postmortem studies, most clinical reports are of female patients [2]. Some reports suggest that gallbladder agenesis is familial [1–6]. Kobacker [3] reported agenesis of the gallbladder in two sisters. Investigation of that family revealed three more sisters with nonfunctioning oral cholecystograms. Sugrue et al. [2] reported ectopia and agenesis of the gallbladder in two sets of twins. These literatures suggested that occurrence of agenesis of the gallbladder might have a genetic connection.

US is the first choice for reliable investigation of the gallbladder and hepatobiliary disease. Generally, when the gallbladder is not detected by US, it is inferred that gallbladder shrinks because of inflammation and because of the presence of gallstones [1]. Consequently, CT, MRCP, and EUS are regarded as subsequent approaches. If additional examinations show no gallbladder without a cystic duct, gallbladder agenesis can be strongly suspected. Nevertheless, final diagnosis for agenesis of gallbladder requires the findings of angiography or surgical operation. In neither case did we perform angiography or surgical operation because both were young females with no symptoms. However, various imaging modalities produced typical findings of agenesis of the gallbladder because we were able to identify the gallbladder and cystic ducts in both cases. In cases of agenesis of the gallbladder, the patients might have some symptoms related to this abnormal condition, such as jaundice attributable to coexisting choledocholithiasis (incidence of 10–30%) [6, 7]. In contrast, if one encounters a patient with choledocholithiasis, then one should approach the case considering the possibility of agenesis of the gallbladder, despite its extreme rarity.

In conclusion, our case strongly suggested that agenesis of the gallbladder would be caused by a genetic abnormality. However, our report just described one case; thus, we thought that it needed more similar cases to elucidate the pathology of agenesis of the gallbladder.
